# Linear Growth in Pediatric Kidney Transplant Population

**DOI:** 10.3389/fped.2020.569616

**Published:** 2020-12-08

**Authors:** Mercedes Lopez-Gonzalez, Marina Munoz, Victor Perez-Beltran, Alejandro Cruz, Romy Gander, Gema Ariceta

**Affiliations:** ^1^Department of Pediatric Nephrology, University Hospital Vall d'Hebron, Barcelona, Spain; ^2^Pediatric Urology and Renal Transplant Unit, Department of Pediatric Surgery, University Hospital Vall d'Hebron, Barcelona, Spain; ^3^Department of Pediatrics, University Autonomous of Barcelona, Barcelona, Spain

**Keywords:** growth, pediatric, kidney transplant, height, growth hormone

## Abstract

**Introduction:** Growth retardation is one of the main complications of chronic kidney disease (CKD) in children and induces a negative impact on quality of life.

**Materials and Methods:** Retrospective analysis of all consecutive patients younger than 18 years old who received a first KT in our center between 2008 and 2018.

**Results:** 95 first KT recipients, median age at KT of 7.83 years. At the time of KT, 65.52% of males and 54.05% females showed normal height. After transplantation, linear growth improved from −1.53 at transplant to −1.37 SDS height at the last visit. We detected a different linear growth pattern according to patient age at KT. Children younger than 3 years old exhibited the most significant growth retardation at baseline and the greatest linear growth over time (−2.29 vs. −1.82 SDS height), whereas catch-up was not observed in older patients. Multivariate analysis showed that use of corticosteroids was negatively related to SDS height at 1 year after transplantation and final SDS height only was positively associated with SDS height at KT. 44.2 and 22.1% patients received rhGH treatment before and after KT. 71.88% patients reached adulthood with normal final height.

**Conclusions:** In our study, pediatric KT recipients exhibited a normal height in more than half of cases at KT and in more than two thirds at the final adult height. Only children younger than 6 years old presented a relevant growth catch-up after KT. Treatment with rhGH was used before and after KT with significant improvement in height.

## Introduction

Growth retardation is one of the main complications of chronic kidney disease (CKD) in children (1, 2). Its etiology is multifactorial, explained by conditions existing prior to kidney transplant (KT) such as primary kidney disease, malnutrition, quality of care, growth deficit at the time of transplantation, dialysis adequacy, or use of recombinant human growth hormone (rhGH) and by others related to KT itself such as living donors, use of corticosteroids, or graft function (1, 2).

According to the North American Pediatric Renal Transplant Cooperative Study (NAPRTCS) and the European Society for Pediatric Nephrology/European Renal Association and European Dialysis and Transplant Association (ESPN/ERA-EDTA), only about half of the patients receiving a KT during childhood reach an adult normal height (SDS >1.88) despite observed improvement in impaired growth management during the last decades (3, 4).

Even KT being the best renal replacement therapy (RRT) modality, its contribution to final height and growth normalization in pediatrics is limited (5). Growth retardation in transplanted children and adolescents overall reflects worse patient clinical status and often is associated with poor medical outcome (6). Furthermore, growth delay induces a negative impact on quality of life (QoL) and social conditions and is linked to a lower level of education and employment. In patients with early-onset renal failure, subjective well-being status correlated with satisfaction with the achieved adult height. Remarkably, 36% of them were not pleased to it (7–9).

Despite the impact of linear growth on pediatric KT recipients QoL, limited real-life data are available. Our aim is to describe the observed growth pattern in a series of pediatric patients who received a KT in our center and to evaluate what factors are associated with patient height, comparing our results with gathered data from large population-based pediatric KT recipient registers.

## Materials and Methods

### Data Collection

For this study, we retrospectively reviewed each patient electronic medical records from the time of KT to the last follow-up in our institution. We included all consecutive patients younger than 18 years old who received a first KT in our center between January 2008 and December 2018. Exclusion criteria were repeated transplantation and graft loss during the first month after KT.

The following patient items were collected: date of birth, gender, primary renal disease, treatment modality before transplantation, date of KT, donor source, use, and duration of rhGH, estimated glomerular filtration rate (eGFR), height (absolute and standard deviation, SDS), date of graft failure (defined as the day of RRT initiation), and its cause. Patient height was assessed at 3 time-points: at the day of KT, 1 year later, and at the final follow-up. eGFR was collected at 1 year after KT and at the end of follow-up. We considered patient final follow-up, the day of last visit with a functional transplant, graft failure, or whenever the patient turned 18 years of age. We recalled information of the duration (months) of treatment with corticosteroids after KT based on local pharmacy electronic prescriptions but could not get the specific cumulative dose used per patient, which represents a limitation of the study.

Height values were expressed as a height standard deviation score for chronological age (SDS) compared with population-based growth reference charts in Spain (10). By consensus, growth deficit was defined as SDS height < −1.88 adjusted for age and gender.

Glomerular filtration rate (GFR) was estimated from plasma creatinine using the adjusted Schwartz formula (11).

### Data Analysis

Statistical analysis was performed using Stata 13.1 (StataCorp, College Station, TX). Variables are expressed as median with interquartile range (IQR) for continuous variables or *n* (percentages) for categorical variables. Student's *t*-test was used for the comparison of continuous variables when normally distributed and Mann–Whitney *U*-test for non-normally distributed variables. Pearson Chi-Square or Fisher's exact test was used for categorical variables. The variations of the SDS throughout the study time (KT, 1 year after KT, final follow-up) in the different age groups (<3, 3–6, 6–12, and >12 years old) were calculated by analysis of the intergroup and intragroup variance and were represented by means of a box plot graph.

Multivariable analysis was conducted through linear mixed model regression analyses adjusted according to the criteria for confounding; variables considered in the adjusted analyses were age, SDS height at KT, rhGH before and after KT, duration of corticosteroids (in months), and eGFR at 1 year and final follow-up after KT. A *p* < 0.05 was considered statistically significant.

## Results

### Baseline Characteristics

We studied 129 consecutive KT performed in children and adolescents <18 years old in our center, between 2008 and 2018. Afterward, we excluded 34 cases due to early graft loss during the first month after transplant (*n* = 11) or repeated transplantation (*n* = 23). Thus, we ended analyzing 95 first KT recipients with a median time of follow-up of 37.8 months (IQR: 17.9–63). 58 (61.05%) were males and 37 (38.95%) females, with a median age at KT of 7.83 years (IQR: 3.3–14.4) years.

Congenital anomalies of the kidney and urinary tract (CAKUT) represented the most common cause (40%) of chronic kidney disease (CKD). Of those, 29% had posterior urethral valves. Further else, 10% of patients had a nephrotic syndrome of genetic origin (4 Finnish type). Other causes of CKD in our cohort were ARPKD (9.5%), nephronophthisis (7.4%), primary hyperoxaluria (2.1%), steroid-resistant nephrotic syndrome (SRNS) (4.2%), cystinosis (3.1%), and Alport syndrome (2.1%).

The most frequent initial RRT modality was hemodialysis (HD) in 52% of the cohort, whereas 31 patients (32.6%) received a preemptive KT. Most patients (*n* = 88; 92.73%) received a deceased donor graft, and 7 (7.37%) a living-related donor one. Further, 2 children with autosomal recessive polycystic kidney disease (ARPKD) underwent a combined liver–kidney transplant (LKT).

The immunosuppressive regimen consisted of an induction treatment with basiliximab and initial triple immunosuppression with tacrolimus, mycophenolate, and corticosteroids. Characteristically in our cohort, median duration of treatment with corticosteroids was 7 months IQR (5–15), with only 11.6% of patients with maintenance of corticosteroids at the end of the follow-up.

In our series, 84 (88.4%) patients had a functioning graft at the end of the follow-up with a median eGFR 62 ml/min/1.73 m^2^. Over time, 11 patients (11.6%) lost their graft due BK nephropathy (*n* = 4), and biopsy-proved transplant glomerulopathy (*n* = 3), acute humoral rejection (*n* = 2), and acute cellular rejection (*n* = 1) (Table 1).

**Table 1:** Baseline characteristics of the study population.

**Variables**	***n* (%)**
**Sex**	
Male	58 (61.05)
Female	37 (38.95)
**Primary renal disease**	
CAKUT	38 (40)
Genetic nephrotic syndrome	10 (10)
ARPKD	9 (9.5)
Nephronophthisis	7 (7.4)
SRNS	4 (4.2)
Primary hyperoxaluria	2 (2.1)
Cystinosis	3 (3.1)
Alport syndrome	2 (2.1)
Miscellaneous	18 (18.9)
Unknown	2 (2.1)
**Preemptive KT**	31 (32.6)
**RRT modality**	
HD	48 (50.5)
PD	16 (16.8)
**Donor source**	
Deceased	88 (92.6)
Living	7 (7.4)
**Age and height at the day of KT**	
Age, years	7.83 (IQR: 3.3–14.4)
Height SDS	−1.53 (IQR: −2.47 to −0.95)
**Graft status at the end of follow-up**	
Failure	11(11.6)
Functioning	84 (88.4)

*CAKUT, congenital abnormalities of kidney and urinary tract; ARPKD, autosomal recessive polycystic kidney disease; SRNS, steroid resistant nephrotic syndrome; KT, kidney transplantation; RRT, renal replacement therapy; HD, hemodialysis; PD, peritoneal dialysis*.

### Linear Growth After Kidney Transplant by Sex

As a group, boys were younger than girls, but age distribution did not differ statistically between gender: 6.8 y (IQR: 3.8–14.4) vs. 9.8 y (IQR: 3.2–14.4) (*p* = 0.29).

At the time of KT, more than half of patients of both sex, 38 males (65.52%) and 20 females (54.05%), showed a normal height (defined as SDS >−1.88, adjusted to age and sex). Further, we observed that, as a group, females were shorter than males, with a median SDS height of −1.76 (IQR: −2.5 to −0.96) and −1.43 (IQR: −2.44 to −0.89), respectively, but without significance (*p* = 0.47).

During the observation period, 32 patients reached adulthood, all of them with a functioning graft. 71.88% of those patients showed a normal final adult height without sex differences. Women (*n* = 16) had a median height of 155 cm (−1.17 SDS) and men (*n* = 16) 167 cm (−1.1 SDS) with similar graft function (eGFR 55.5 vs. 58.3 ml/min/1.73 m^2^, respectively).

### Linear Growth After Kidney Transplant by Age

We observed significant improvement of linear growth after transplantation in the whole series over time, from SDS height −1.53 (IQR: −2.47 to −0.95) at the day of transplant to a SDS height −1.37 (IQR: −1.92 to −0.61) at the last visit (*p* = 0.039), without differences between sex. Specifically, growth improvement occurred later than 1 year, as we did not find growth catch-up during the first year after transplantation [SDS height −1.46 (IQR: −2.33 to −0.85)], compared to baseline.

**Figure 1:**
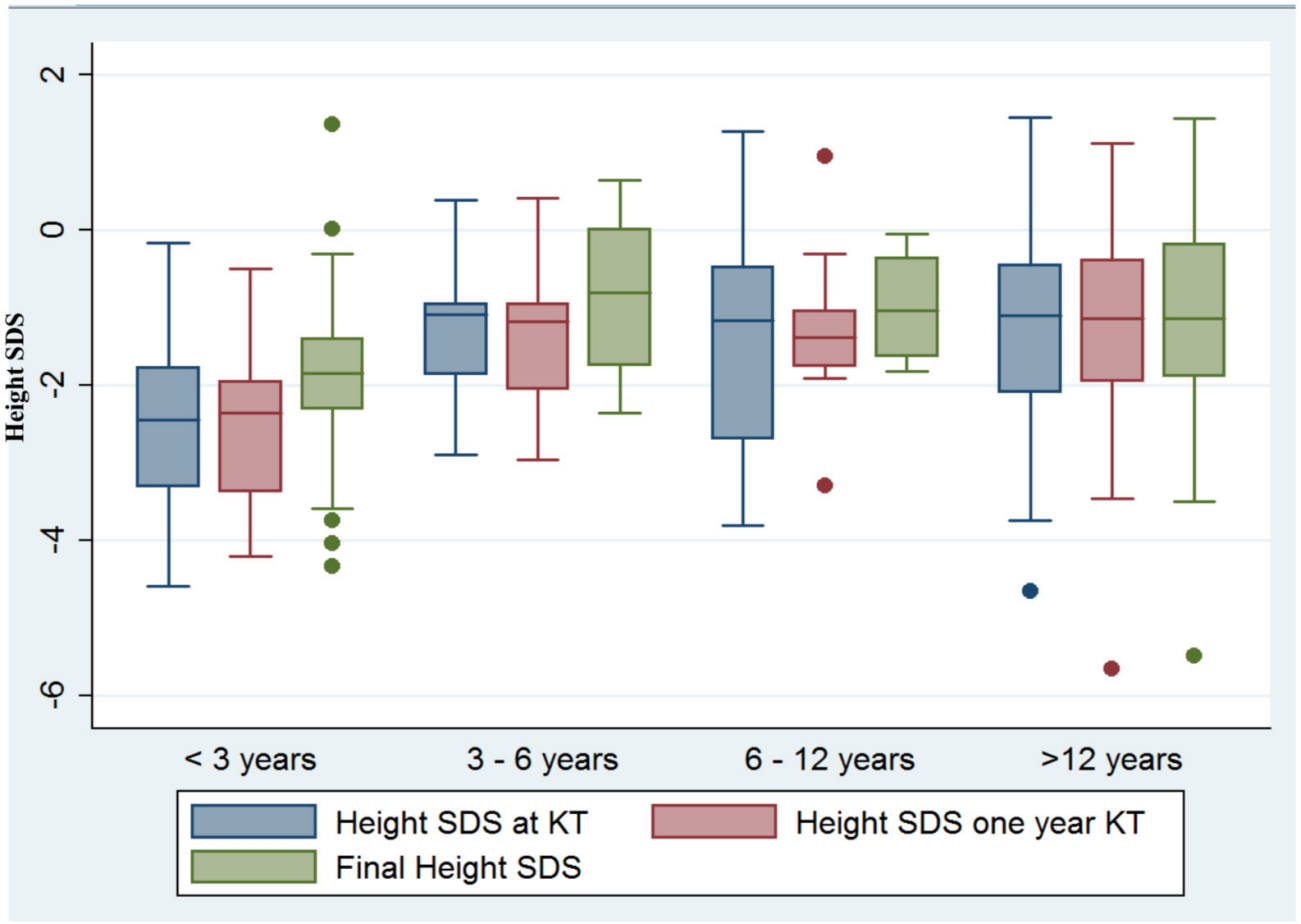
Linear growth after KT according to patient age at the day of transplant. Children younger than 3 years old exhibited the most significant growth retardation at baseline and the greatest linear growth over time (−2.29 SDS height vs. −1.82 SDS height, *p* = 0.02), and also children from 3 to 6 years of age improved significantly (SDS height −1.26 to −0.74, *p* = 0.04), whereas catch-up was not observed in older patients.

Remarkably we detected a different linear growth pattern after transplantation according to patient age at KT (Figure 1). Thus, children younger than 3 years old (*n* = 16; 16.84%) showed the most significant growth retardation (−2.29 SDS height) at baseline, but the greatest linear growth over time, achieving a normal stature (SDS height −1.82) at the last follow-up visit (*p* = 0.02). Further, patients from 3 to 6 years old also experienced significant growth catch-up from SDS height −1.26 to −0.74 at the end of the follow-up, but in less extent than the youngest ones. Older patients got a normal stature at the last measurement after transplant as well but did not experience catch-up either: from SDS height SDS −0.98 to −0.82 in children from 6 to 12 years old (*p* = 0.05), whereas in adolescents (patients >12 years old), SDS-height after KT worsened from −1.28 to −1.38 SDS, but without statistical significance and within the normal range.

### Treatment With Recombinant Human Growth Hormone

Forty-four patients (44.2%) received recombinant human growth hormone (rhGH) before KT, 50% of boys and 40% of girls. The median age at the beginning of rhGH was 2.54 years old, without significant differences by sex.

Children treated with rhGH achieved similar SDS height at time of transplantation compared to those without hormone treatment (−1.8 vs. −1.5) (*p* = 0.30).

As short stature remained after KT, 22 patients (23.1%) received treatment with rhGH during post-transplant follow-up. Thirteen of them had also received rhGH prior to transplant (59%). Those patients exhibited severe growth retardation at KT baseline (height SDS −2.42) which had worsened by the time of rhGH initiation (height SDS −2.94). Outcome became positive and clearly growth improvement occurred in those children who achieved a normal height (SDS height −1.8) at the end of follow-up. Fifteen out of those 22 patients (68.2%) had a functioning graft (eGFR 62.7 ml/min/1.73 m^2^).

The rate of rhGH prescription after transplant turned out to be more than 3-fold times frequent in patients suffering graft failure compared to those with a functioning graft (63.6 vs. 16.7%), differences not observed before KT (45.4 vs. 46.4%). At the end of follow-up, the first subgroup achieved slightly less stature (height SDS −1.55 vs. −1.22) (Table 2).

**Table 2:** Patient characteristics according to graft status.

	**Functional grafts (*n* = 84)**	**Graft failure (*n* = 11)**
Age at KT (years)	8.92	3.25
Height (SDS) at KT	−1.39	−2.7
Preemptive KT (%)	27 (32.1)	4 (36.4)
rhGH before KT (%)	39 (46.4)	5 (45.4)
rhGH after KT (%)	14 (16.7)	7 (63.6)
eGFR at first year after KT^*^	73.4	70.4
eGFR at final follow-up^*^	62	–
Height (SDS) at final follow-up	−1.22	−1.55

*KT, kidney transplantation; eGFR, estimated glomerular filtration rate*.

* *eGFR = ml/min/1.73 m^2^*.

### Other Factors Related to Growth After Transplantation

At transplant baseline, observed SDS-height was better in children who received a preemptive KT (SDS −1.15) than in those after chronic dialysis (SDS −1.7) (*p* ≤ 0.07). However, no differences in final height were found according to sex, preemptive KT vs. dialysis or primary renal disease (CAKUT vs. no CAKUT).

In order to evaluate each factor impact on growth, we performed a multivariate regression analysis including the following variables: age, SDS height at KT, rhGH before and after KT, duration of corticosteroids (in months), and eGFR at 1 year and final follow-up after KT. SDS height at KT and the use of corticosteroids after transplantation were found to be positively and negatively related to SDS height at 1 year after transplantation (*p* ≤ 0.01 and *p* ≤ 0.04, respectively) (Table 3). However, final SDS height only was positively associated with SDS height at the day of transplantation (*p* ≤ 0.01) and negatively by the use rhGH after KT (*p* ≤ 0.05), without impact of corticosteroids treatment (Table 4).

**Table 3:** Multivariate regression analysis for factors influencing SDS height one year after KT.

**Variables**	**Coefficient**	**95% CI**	***P*-value**
Age at KT	−0.04	−0.03 to 0.02	0.70
Height (SDS) at KT	0.86	0.76 to 0.96	0.01
rhGH before KT	−0.07	−0.34 to 0.20	0.62
rhGH after KT	−0.44	−0.77 to −0.11	0.09
Corticosteroids	−0.01	−0.02 to 0.01	0.04
eGFR 1 year after KT	0.01	−0.01 to 0.02	0.76

*KT, kidney transplantation; eGFR, estimated glomerular filtration rate*.

**Table 4:** Multivariate regression analysis for factors influencing SDS final height.

**Variables**	**Coefficient**	**95% CI**	***P*-value**
Age at KT	−0.03	−0.08 to 0.01	0.17
Height SDS at KT	0.60	0.42 to 0.78	0.01
rhGH before KT	−0.03	−0.50 to 0.44	0.89
rhGH after KT	−0.58	−1.16 to 0.01	0.05
Corticosteroids	−0.01	−0.02 to 0.01	0.47
eGFR final follow-up	0.01	−0.01 to 0.02	0.57

*KT, kidney transplantation; eGFR, estimated glomerular filtration rate*.

## Discussion

Growth retardation is one of the most important adverse events observed in children suffering from CKD, with significant implications in perceived quality of life during childhood and adulthood. Despite of KT being the best RRT modality, its contribution to final height and growth normalization is limited, even with a functioning graft.

There is scarce information about linear growth pattern in pediatric patients who received a KT. In this case series, we observed a better Z score of height at the day of KT and at the last follow-up visit (−1.53 and −1.16 SDS, respectively) compared to other similar published cohorts published (3, 4). Further, we observed a normal stature in more than half of children at the time of KT.

According to the ESPN/ERA-EDTA registry report over the past 25 years in Europe, KT had a mild positive impact on linear growth (−1.87 and −1.77 SDS before and after KT) and only 55.1% of children who received a kidney graft achieved a normal height (>−1.88 SDS) at the end of follow-up (4). Similarly, in North America, KT did not produce relevant changes in height in pediatric patients according to the NAPRTCS 2014 annual report which described a Z score of −1.73 SDS at the day of KT and −1.77 SDS at the last visit (3).

Further, data analysis from European and North American registries suggests that a functioning graft by itself does not correct severe growth retardation seeing in some children with CKD. Indeed, more aggressive management before and after transplantation is advocated: early detection of growth failure, close monitoring of height and growth velocity, correction of hydro-electrolyte disturbances, mineral and bone disorders, and considering using rhGH when every previous factor is corrected (12).

The fact that in our center KT recipients were taller than described could be explained by the large percentage of preemptive KT performed—a finding associated with better stature—in more than one third of patients, meaning early access to KT in our program in a high proportion of patients, as KT is best RRT modality. Further, in our center it is remarkable the large percentage of patients treated with rhGH, before and after transplantation (44.2 and 23%, respectively), in comparison with other series published, While an ESPN/ERA-EDTA report in 2016 revealed that treatment with rhGH was underused both in patients with growth failure in CKD and, especially, after transplantation. Only 24% of short children in dialysis and 7.6% of transplanted patients received it in Europe (13).

At least part of the low rhGH prescription rate may be explained by the fact that initially, treatment with rhGH in pediatric KT recipients was believed to be associated with increased graft loss risk or graft function impairment (14, 15). However, more recent studies have vastly demonstrated that those hypotheses were not true (16–20). For that reason, currently, treatment with rhGH in pediatric transplanted patients with growth retardation is thought to be safe, and its utilization should be promoted after KT if applicable, in order to achieve normal height. Our results support this concept as well.

In the current study, multivariate regression analysis showed that the use of rhGH has a negative impact on height. Hormone treatment is considered in this case as a confounding factor, due to its use, before and after transplantation was reserved, for patients with greater growth retardation.

As mentioned above, improvement in linear growth after transplantation is limited; nevertheless, in this cohort, the observed pattern of growth after transplantation was not uniform. Children younger than 6 years old were the only ones who experienced significant growth catch up after KT (*p* = 0.04). This effect was more remarkable among the youngest children (*p* = 0.02) despite that they had the most severe growth deficiency. Reversely, teenagers older than 12 years old demonstrated either no increase or even a decrease in Z-height score. These findings replicate those previously described in literature, mainly in large series such as NAPRTCS and ESPN/ERA-EDTA (3, 4).

However, some reports from single centers also had observed a catch-up growth in pubertal and adolescents patients (21, 22). Delayed onset and short duration of puberty observed in CKD patients has a negative impact on growth in adolescents receiving a KT (2). A recent study about treatment with rhGH in males transplanted during late puberty showed improvement in final height (23). These results could make us consider rhGH prescription in KT adolescents in an attempt to mitigate the impact of short puberty in growth spurt and adult height.

When growth was analyzed by gender, we found that girls had worse stature compared to boys, either at the day of KT (Z score height −1.76 SDS and −1.43 SDS) and at the last visit later on (−1.27 SDS and −1.15 SDS, respectively). Despite lack of significance in our series, those findings were comparable with those reported by the European registry (4) but differed from NAPRTCS and other single-center reports (3, 24).

One of the reasons for those differences is based on previously documented gender disparities in access to pediatric kidney transplantation, particularly preemptive KT. Both in Europe and North America, girls were less likely to receive a preemptive KT than boys (25, 26). Preemptive transplantation or shorter period of time on dialysis had been previously described as factors related with better height prior to transplant and after it (2, 22, 24).

Another argument may be related to a reduced use of rhGH in females compared to males. In our study, we observed less rhGH prescription in girls than boys (40.54 vs. 50%, respectively), despite shorter stature in females but without significance. Further prospective studies are required to better evaluate if there is a gender difference in rhGH use.

Graft function, represented as GFR, is a widely demonstrated factor associated with growth improvement both in CKD and KT children (1, 4, 21, 27). We found that in this series, more frequent rhGH indication in patients with graft dysfunction prevented from additional height worsening. Indeed, those patients only showed a slightly reduced Z-height at the end of the follow-up compared to those with functioning grafts (−1.55 vs. −1.22 SDS).

Although living donor grafts (2, 4, 28) has been identified as a contributor factor to improve growth after transplantation, this analysis is limited in our cohort as only 7 related living donor KT were performed during the period of observation, a situation that is changing recently with increased living donor transplants.

It is widely known that corticosteroid avoidance or withdrawal (4, 29–32) is a main contributor factor for growth after KT. However, one important limitation of our study is lack of precise corticosteroid dosage used due the retrospective nature of the study, which precluded extensive analysis of this variable. However, we could account the length of corticosteroid use in our series, and we demonstrated its negative impact on patient growth during the first year after KT (*p* ≤ 0.05), but not any significant impact on patient height at the final follow-up. These observations are consistent with the active corticosteroid treatment avoidance regimen of our program, with very few individuals receiving corticosteroids beyond 7 months after KT.

Remarkably, in our center, 71.88% of patients who reached adulthood showed a normal final height without relevant gender differences (women 155 cm, −1.17 SDS vs. men 167 cm −1.1 SDS). These data were similar to those obtained in the NAPRTCS report as well as in multiple studies from single centers in Germany, Sweden, Australia, and Spain, ranging from 68 to 75% of patients reaching a normal height at adulthood (3, 21, 24, 33, 34). All these results show a clear improvement from those observed in older reports (23–38%) (2, 35).

The greatest strength in our study was the access to preemptive KT, more frequent rhGH use in the population of CKD, and KT recipients having achieved better stature outcome. We should mention some weaknesses in our work. First, it was a retrospective study in a single center with a limited sample size. Also, other determinant factors for growth, such as nutritional status, anemia, hypertension, living donor grafts, and corticosteroid therapy as mentioned were not analyze in detail.

In conclusion, our study showed that in a single center, pediatric KT recipients achieved normal height in more than half of cases at the time of transplantation and in more than two thirds at the final adult height. No sex differences were found. Only children younger than 6 years old presented a relevant growth catch-up after KT, whereas older patients continued growing in a steady pattern. Treatment with rhGH was used before and after KT with significant improvement in height. Therefore, we suggest more interventional growth retardation treatment in CKD and KT patients, with more frequent use of rhGH.

## Data Availability Statement

The original contributions presented in the study are included in the article/supplementary material, further inquiries can be directed to the corresponding author/s.

## Ethics Statement

The studies involving human participants were reviewed and approved by Vall d'Hebron Hospital. Written informed consent to participate in this study was provided by the participants' legal guardian/next of kin.

## Author Contributions

ML-G and GA conceived and designed the project. MM, VP-B, and AC recruited the patients. ML-G, RG, and MM collected the data and conducted the data analysis. ML-G wrote the first draft of the manuscript and all authors, especially GA, contributed to the critical appraisal of the manuscript.

## Conflict of Interest

The authors declare that the research was conducted in the absence of any commercial or financial relationships that could be construed as a potential conflict of interest.
